# Egg White Hydrolysate Retains the Nutritional Value of Proteins and Is Quickly Absorbed in Rats

**DOI:** 10.1155/2019/5475302

**Published:** 2019-08-27

**Authors:** Ryosuke Matsuoka, Hitoshi Kurihara, Noriaki Nishijima, Yoshifumi Oda, Akihiro Handa

**Affiliations:** R & D Division, Kewpie Corporation, Sengawa Kewport, 2-5-7, Sengawa-cho, Chofu-shi, Tokyo 182-0002, Japan

## Abstract

Egg white protein has a high net protein utilisation, with a score of 100 in the amino acid rating system. Although the enzymatic breakdown of egg white yields hydrolysates that are rapidly absorbed and various physiological activities can be expected from them, flavouring egg white to meet taste requirements as a food has been a difficult challenge. Herein, we developed a high-molecular-weight egg white hydrolysate and compared the absorption rate and nutritional value of the hydrolysate with those of egg white proteins obtained from raw materials, whey proteins, and hydrolysates, also known as high-quality proteins. The absorption rate of egg white hydrolysates was faster than that of egg white and whey proteins in portal vein cannulated rats, and their bioavailability values were higher than those of whey proteins and hydrolysates. According to the protein digestibility-corrected amino acid score and digestible indispensable amino acid score, the scores for egg white hydrolysates were equivalent to those of egg white and whey proteins but higher than those of whey hydrolysates. Our results show that egg white hydrolysates maintain the nutritional value of egg whites and are rapidly absorbed by the body.

## 1. Introduction

Egg whites are known to be a source of high-quality proteins, with an amino acid score of 100, which is equivalent to that of milk and soybeans [1]. A previous study has shown that, despite equivalent amino acid scores and digestion-absorption rates, the net protein utilisation (NPU) value for egg white proteins, whether cooked or uncooked, is higher than that of whey and soybean proteins [2]. The antioxidant effect of low-molecular-weight egg white hydrolysates generated from egg whites has also been reported [3]. In addition to the nutritional properties of proteins, it is expected that their new physiological functions will be discovered.

One challenge associated with egg white hydrolysates is meeting food taste requirements. During the conversion of high-molecular-weight hydrolysates to low-molecular-weight hydrolysates, the hydrolysates become bitter. Another general characteristic of the hydrolysates is that compared with proteins, they have lower molecular weight, which results in faster absorption; however, a hydrolysate that retains high molecular weight has slower absorption rates and does not function as a hydrolysate.

In a previous study, no difference was observed when comparing NPU between egg yolk hydrolysates and proteins obtained from raw materials [4]; however, because there have been no previously reported comparisons using egg whites, we compared the NPU of egg white hydrolysates with that of egg white proteins.

Additionally, because the amino acid scores have been based on the chemical analysis of the amino acid composition of the product, digestion/absorption has not been considered; therefore, amino acid scores (protein digestibility-corrected amino acid score (PDCAAS) and digestible indispensable amino acid score (DIAAS)) that consider digestibility/absorption have been proposed [5]. However, although an *in vitro* evaluation of the DIAAS in egg white proteins has been reported, there have been no *in vivo* reports [6]. Considering the above, in this study, we aimed to develop a high-molecular-weight hydrolysate from egg whites with minimal bitterness, compare the portal blood absorption of egg white hydrolysates with that of whey hydrolysates, and calculate the DIAAS in both egg white proteins and hydrolysates.

## 2. Materials and Methods

### 2.1. Materials

Whey and egg white proteins were obtained from Kewpie Egg Corporation (Tokyo, Japan). Milk whey hydrolysates (8390 WPH HYDROLYZEDR) and egg white hydrolysates (EP-3R) were obtained from Hilmar Ingredients (Hilmar, CA, USA) and Henningsen Foods Inc. (David City, NE, USA), respectively. Protein content was analysed using the Dumas method for protein determination with a factor of 6.25 × total nitrogen (N) for determining total protein [7]. The protein content for each of the samples was as follows: whey protein, 80.7 g/100 g; whey hydrolysate, 74.8 g/100 g; egg white protein, 83.3 g/100 g; and egg white hydrolysate, 82.6 g/100 g. The molecular weights of whey and egg white hydrolysates were approximately 500 and 2,500 Da, respectively.  Table 1 provides the amino acid composition of each sample. Amino acid composition was analysed at the Japan Food Research Laboratories (JFRL).

### 2.2. Amino Acid Absorption Values Using a Portal Vein Cannulation Rat Model

Twenty Sprague Dawley (SD) rats weighing 201–225 g each were obtained from SLC, Inc. (Shizuoka, Japan), for portal vein cannulation [8]. Before administering test samples to the rats, blood samples were taken after the rats were fasted overnight. The rats were separated into four groups for oral administration of 1 g/kg of one of the following: milk whey protein, milk whey hydrolysate, egg white protein, or egg white hydrolysate. After 10, 30, 60, and 120 min, blood samples were collected again using a heparin-containing syringe and centrifuged at 8000 rpm for 15 min.

Amino acid content was analysed using the L-Amino Acid Quantitation Kit (BioVision Inc., Milpitas, CA, USA) following treatment with trichloroacetic acid to remove proteins from the plasma [9]. This experiment was conducted under the guidelines of the Act on Welfare and Management of Animals (Act No. 105) and Notification No. 6 of the Government of Japan. Animal experiments were also conducted at the Research and Development Division of Kewpie Corporation in accordance with the regulations for animal testing (Approval No. 17-01). Animal studies were conducted on 28 and 29 December 2016.

### 2.3. Evaluation of Digestion/Absorption and Bioavailability Rates

Thirty SD rats weighing 202–228 g each were housed in metabolic cages (Toyoriko Co., Ltd, Tokyo, Japan) under the following settings: light cycle, 8:00 to 20:00; temperature, 23 ± 1°C; and humidity, 50 ± 2%.

Rat feed was prepared according to the American Institute of Nutrition Rodent Diet (AIN-76) [10]. The composition was as follows: 10% or 0% proteins (milk whey protein, milk whey hydrolysate, egg white protein, and egg white hydrolysate), 15% *β*-corn starch, 5% cellulose, 3.5% mineral mix (AIN-76), 1% vitamin mix (AIN-76), 5% corn oil, 0.2% choline bitartrate, and 0.3% titanium dioxide and sucrose added to bring the total to 100%. The rats were divided into four groups according to diet (milk whey protein, milk whey hydrolysate, egg white protein, and egg white hydrolysate or 0% protein) with the group receiving 0% protein used as the control to calculate background metabolic N. Titanium dioxide was added as a marker of undigested feed. On the basis of a previous study, the current study was conducted using the AIN-76 formulation with 10% protein [11, 12]. The basis for setting the 10% protein level was to more easily determine protein bioavailability at lower protein levels and compare the data with those of previous studies.

The five groups were observed by pair-feeding over a 10 d period. The rats were free to consume distilled water. Faeces and urine were collected over the 5 days before the end of the study period to measure N concentration and calculate the digestion/absorption ratio, NPU, and PDCAAS. The amount of N excreted in the faeces and urine in the group fed a diet with 0% protein was 16.8 ± 0.7 mg/5 d (metabolic faecal N) and 26.2 ± 1.6 mg/5 mg (metabolic urinary N), respectively.

This experiment was conducted under the guidelines of the Act on Welfare and Management of Animals (Act No. 105) and Notification No. 6 of the Government of Japan and carried out on 5–22 December 2017 at the Research and Development Division of Kewpie Corporation (Approval No. 17-05).

Protein levels in faeces and urine were determined using the method of Dumas using 6.25 × total N to determine total protein [7]. The protein efficiency ratio, digestion/absorption ratio, and NPU were calculated using the following formulas [13]:

  digestibility = (N intake (faecal N − metabolic faecal N))/N intake × 100
  NPU = digestibility − (N intake − (urine N − metabolic urine N))/N intake × 100

### 2.4. Calculation of PDCAAS and DIAAS

PDCASS was calculated on the basis of the amino acid score of the feed multiplied by the digestibility percentage. Amino acid scores for a child ≥6 years old were used as a reference. Values >1 were recorded as 1 [5].

The DIAAS method for assessing protein quality is based on the titanium content in food and within the ileum, and the ileal absorption values of the essential amino acids were calculated using reference amino acid scores for a child ≥3 years old [5]. Amino acid analyses in food and the ileum were conducted at JFRL, a general incorporated foundation, and Japan Special Animal Diagnostic Services Ltd. Titanium dioxide levels were determined according to the method of Short et al. [14].

### 2.5. Statistical Analyses

The study results are displayed as mean ± standard deviation. Statistical analyses were performed using one-way analysis of variance with Tukey's test when significant differences were observed among datasets. A *p* value <0.05 was considered significant. Statistical analyses were conducted using SPSS II for Windows (SPSS Japan Inc., Tokyo, Japan).

## 3. Results

### 3.1. Plasma Amino Acid Levels in Rats Administered Egg White or Milk Whey Protein or Hydrolysates

**Figure 1:**
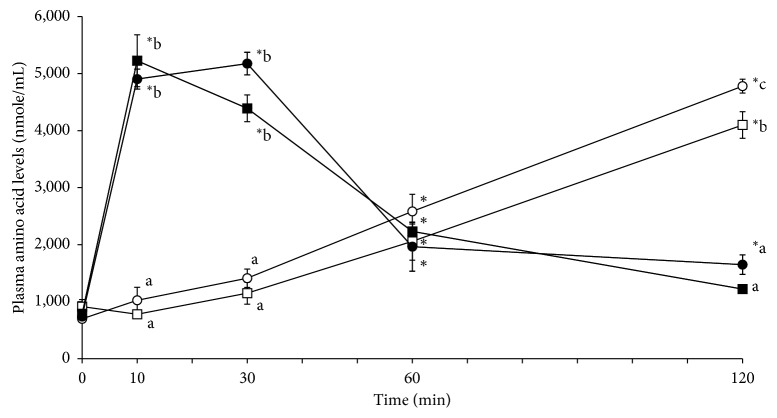
Changes in plasma amino acid levels in portal vein cannulated rats: the rate of amino acid absorption in portal vein cannulated rats administered milk whey protein, milk whey hydrolysate, egg white protein, or egg white hydrolysate. □: milk whey protein; ■: milk whey hydrolysate; 〇: egg white protein; •: egg white hydrolysate. Data are mean ± SE of 6 rats. Different letters show significant difference at *p* < 0.05 by Tukey's test.^∗^*p* < 0.05 vs. 0 minutes by the Dunnett test.

Figure 1 shows the amino acid concentrations in the portal blood of rats according to protein and hydrolysate intake. The amino acid concentrations in rats 10 and 30 min after being fed egg white or whey hydrolysate were significantly higher than those in rats fed egg white or whey protein. No significant difference in amino acid concentration in the blood was observed between rats fed either egg white or whey hydrolysate. After 120 min, the amino acid concentrations in the blood of rats fed egg white or whey protein were significantly higher than those in rats fed egg white or whey hydrolysate. Moreover, amino acid concentrations in the blood were significantly higher in rats fed egg white protein than those in rats fed whey protein. No significant difference was observed between rats fed egg white hydrolysates and those fed whey hydrolysates.

### 3.2. Growth Parameters

Table 2 shows the growth parameters used for the rats. In the four groups receiving a diet containing proteins, there were no differences observed in weight gain increase, food intake, or protein efficiency.

### 3.3. Nitrogen Content in Faeces and Urine

Table 3 shows faecal and urine output and the N content in both faeces and urine. No significant differences in urine output, faecal output, or faecal and urine N were observed among the four groups fed a protein-containing diet. Faecal N content values were significantly lower in the groups fed egg white proteins or egg white hydrolysates than in the groups fed whey proteins and whey hydrolysates. No significant differences were observed in the output of N in the urine between the egg white protein and egg white hydrolysate groups and between the whey protein and whey hydrolysate groups.

### 3.4. Digestibility and NPU

**Figure 2:**
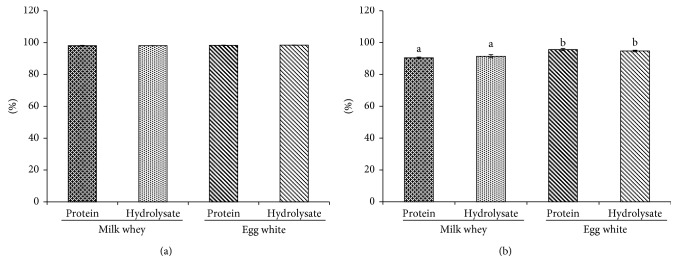
Digestibility (a) and net protein utilisation (b) of whey or egg white protein or hydrolysate. Data are mean ± SE of 6 rats. Different letters show significant difference at *p* < 0.05 by Tukey's test.

Protein digestion/absorption and NPU rates are shown in Figure 2. The results show no significant differences among the four groups with respect to digestion/absorption rates; however, protein utilisation rates in the groups fed egg white protein and egg white hydrolysate were 94.6 ± 0.7 and 94.7 ± 0.5, respectively, which were significantly higher than those in the whey protein group (90.4 ± 0.5) and whey hydrolysate group (91.4 ± 1.0). There was no significant difference observed between the groups fed egg white protein and egg white hydrolysate and those fed whey protein and whey hydrolysate.

### 3.5. Essential Amino Acid Absorption in the Ileum

The absorption rate of essential amino acids in the ileum was ≥98.5% in the whey protein, whey hydrolysate, egg white protein, and egg white hydrolysate groups (Table 4).

### 3.6. PDCAAS and DIAAS

The PDCAAS value was 1.0 in the whey protein, egg white protein, and egg white hydrolysate groups, which was higher than that in the whey hydrolysate group (0.922). The DIAAS value was 1.45 for both the whey protein and egg white protein groups. Although the value in the egg white hydrolysate group was low (1.43), it was higher than that in the whey hydrolysate group (1.25) (Table 5).

## 4. Discussion

The results of this study showed that amino acids were absorbed in portal blood faster in groups fed egg white or whey hydrolysate than in those fed egg white or whey protein (Figure 1). This phenomenon might be explained by the lower molecular weight of hydrolysates. In contrast, the absorption rates of whey hydrolysates with a molecular weight of 500 were approximately equivalent to those of egg white hydrolysates with a molecular weight of 2500. Although the underlying reason for this observation cannot be explained, our results showed that egg white proteins are absorbed faster than whey proteins (Figure 1). One possible explanation is the faster ileal absorption of methionine, phenylalanine, and other essential amino acids after ingesting egg white hydrolysates compared with that after ingesting whey hydrolysates (Table 4); however, the results of this study did not determine the reason for the faster absorption rates despite the higher molecular weight of the egg white hydrolysate. Factors other than greater absorption of essential amino acids, such as amino acid sequences in proteins or amino acid composition, might also be involved.

Our results show that NPU after ingesting egg white proteins was significantly higher than that after ingesting whey proteins (Figure 2). These results are consistent with those of a previous report [2]. We also showed that NPU after ingesting egg white hydrolysates was higher than that after ingesting whey hydrolysates. One reason for this might be that the amounts of cysteine, methionine, and other sulphur-containing amino acids in egg white hydrolysates are higher than those in whey hydrolysates (Table 1). Furthermore, it has been reported that there is no difference in NPU between ingestion of whey proteins and ingestion of whey hydrolysates, which is consistent with our findings [15].

Although the DIAAS for whey proteins has been previously reported, one report was based on the results of an experiment using pigs [16] and another was based on using rats with a reference score for those 0.5–3 years old [17]. Because the age of the rats used in our study was equivalent to that of a human ≥3 years old, we used a reference score for those ≥3 years old to extrapolate the rat results to humans. In addition, we used a reference amino acid score for a child ≥3 years old because our developmental activities were based on the assumption that the health effects of egg white proteins and hydrolysates are observed in a child within that age range.

Proteins are important nutrients for human life because they serve as raw material for muscle, enzyme, and hormones. Moreover, the body cannot produce essential amino acids; therefore, they must be obtained from food. Of all the essential amino acids, a distinctive feature of egg white proteins is that they contain many branched-chain and sulphur-containing amino acids. Previous studies have reported that proteins in the body increase in response to ingesting egg white proteins [18]. In addition, ingesting egg white proteins after exercise increases mass and strength of muscles [19]. Health effects of egg white proteins also include lower serum LDL-cholesterol levels [20] and reduced visceral fat [21]. On the basis of higher NPU, several physiological functions can be expected.

A distinctive feature of enzymatic hydrolysis of egg white proteins is the yield of hydrolysates with antioxidant properties, which have been reported to have antifatigue activities that are stronger than those of egg white proteins [22].

The results of this study show that, without modification, the egg white hydrolysate EP-3 has nutritive value with a high absorption rate. Since EP-3 is equivalent in nutritional value to egg white proteins, it can be expected to be a nutritional food source.

Because we did not evaluate whether this egg white hydrolysate has physiolosical activities in this experiment, additional studies are needed to clarify the health effects of this hydrolysate.

## Figures and Tables

**Table 1:** Amino acid composition of protein or hydrolysate of milk whey or egg white (g/100 g).

	Milk whey		Egg white	
	Protein	Hydrolysate	Protein	Hydrolysate
Ile	4.88	5.15	4.52	4.43
Leu	10.9	8.11	7.44	7.25
Lys	8.84	7.20	6.04	5.87
Met	1.87	1.53	3.41	3.29
Cys	2.42	1.76	2.48	2.44
Phe	3.22	2.41	5.17	5.05
Tyr	3.25	2.02	3.58	3.44
Thr	4.63	5.41	4.01	3.85
Trp	1.94	1.40	1.27	1.27
Val	4.72	4.59	5.99	5.83
His	2.05	1.50	2.04	1.97
Arg	2.58	1.82	5.05	4.91
Ala	4.37	3.88	5.21	5.09
Asp	9.98	8.21	9.15	8.80
Gul	14.6	13.2	11.6	11.4
Gly	1.80	1.37	3.13	3.04
Pro	4.24	4.69	3.30	3.18
Ser	4.15	3.88	5.88	5.74

**Table 2:** Growth variables in rats fed a diet containing protein or hydrolysate of milk whey or egg white.

	Milk whey		Egg white	
	Protein	Hydrolysate	Protein	Hydrolysate
Initial body weight (g)	277 ± 6	277 ± 4	277 ± 3	277 ± 3
Final body weight (g)	317 ± 12	320 ± 4	333 ± 3	336 ± 2
Body weight gain (g/day)	3.98 ± 0.11	4.22 ± 0.14	5.55 ± 0.18	5.84 ± 0.21
Food consumption (g/day)	19.3 ± 0.9	20.3 ± 0.0	20.2 ± 0.0	20.2 ± 0.1
Food efficiency^*∗*^	0.196 ± 0.055	0.208 ± 0.007	0.274 ± 0.008	0.288 ± 0.010

Data are mean ± standard error (SE) of six rats. ^*∗*^Body weight gain (g/day)/food consumption (g/day).

**Table 3:** Faecal and urine nitrogen content in rats fed a diet containing protein or hydrolysate of milk whey or egg white.

	Milk whey		Egg white	
	Protein	Hydrolysate	Protein	Hydrolysate
Faecal weight (dry·g/5 days)	8.72 ± 0.18	8.58 ± 0.15	8.44 ± 0.11	8.50 ± 0.07
Urine volume (mL/5 days)	73.6 ± 14.1	68.5 ± 8.7	70.4 ± 11.4	57.2 ± 6.2
Faecal nitrogen (mg/5 days)	43.5 ± 0.9	46.6 ± 1.0	44.1 ± 0.9	41.4 ± 1.9
Urine nitrogen (mg/5 days)	135 ± 8^a^	133 ± 16^a^	84.3 ± 11.5^b^	85.8 ± 7.3^b^

Data are mean ± SE of six rats. Superscript letters indicate a significant difference (Tukey's test, *p* < 0.05).

**Table 4:** Amino acid absorption (%) in the ileum of rats fed protein or hydrolysate of milk whey or egg white.

	Milk whey		Egg white	
	Protein	Hydrolysate	Protein	Hydrolysate
Ile	99.4	99.3	99.3	99.5
Leu	99.4	99.4	99.4	99.5
Lys	99.8	99.8	99.7	99.8
Met	99.4	99.2	99.7	99.7
Cys	100	100	100	100
Phe	99.3	98.9	98.5	99.6
Tyr	99.7	99.5	99.7	99.8
Thr	99.3	99.3	99.1	99.1
Trp	99.4	99.0	98.9	99.2
Val	99.1	99.0	99.2	99.4
His	99.7	99.5	99.7	99.7

**Table 5:** PDCAAS and DIAAS in rats fed protein or hydrolysate of milk whey or egg white.

	Milk whey		Egg white protein	
	Protein	Hydrolysate	Protein	Hydrolysate
PDCAAS	1.00	0.922	1.00	1.00
DIAAS	1.45	1.25	1.45	1.43

## Data Availability

All data generated or analysed during this study are included in this published article.

## Abbreviations

NPU: Net protein utilisation
PDCAAS: Protein digestibility-corrected amino acid score
DIAAS: Digestible indispensable amino acid score
SD: Sprague Dawley
AIN: American Institute of Nutrition.
